# Anti-S100A4 antibody administration alleviates bronchial epithelial–mesenchymal transition in asthmatic mice

**DOI:** 10.1515/med-2022-0622

**Published:** 2023-10-17

**Authors:** Shuang Liu, Min Liu, Jinnan Zhong, Shi Chen, Ziming Wang, Xiaoyan Gao, Fajiu Li

**Affiliations:** Department of Respiratory and Critical Care Medicine, Affiliated Hospital of Jianghan University, Wuhan 430000, Hubei, China; Department of Respiratory and Critical Care Medicine, Affiliated Hospital of Jianghan University, No. 168, Hong Kong Road, Jiang’an District, Wuhan 430000, Hubei, China

**Keywords:** S100A4, asthma, epithelial–mesenchymal transition, airway remodeling

## Abstract

We elucidated the effect of S100A4 on airway remodeling by regulating airway inflammation and epithelial–mesenchymal transition (EMT) in mouse models of asthma. Asthmatic mouse models were established by sensitization and challenged with ovalbumin (OVA). Anti-S100A4 antibody or control IgG antibody was administered daily before the OVA challenge. After the last challenge, airway inflammation and airway hyperresponsiveness were measured; lung tissues and bronchoalveolar lavage fluid (BALF) were harvested. Lung tissue sections were stained and evaluated for pathological changes. Levels of inflammatory cytokines were measured using ELISA. Levels of S100A4 and EMT markers were determined via western blotting analysis. Human bronchial epithelial cells were stimulated with 100 mg/mL house dust mites (HDMs) to evaluate the effect of S100A4 downregulation on EMT *in vitro*. S100A4 was increased in lung tissues and BALF from asthmatic mice. The asthmatic mice presented airway hyperresponsiveness, airway inflammation, and airway remodeling. After anti-S100A4 antibody administration, pathophysiological signs, including airway hyperresponsiveness and increased infiltration of inflammatory cells, were attenuated. Additionally, anti-S100A4 administration downregulated vimentin and α-SMA expression and upregulated E-cadherin expression in OVA-challenged mice. S100A4 downregulation also inhibited EMT process in HDM-stimulated 16HBE cells. Anti-S100A4 antibody administration alters airway remodeling by preventing EMT in mouse models of asthma.

## 1 Introduction

Asthma is a common airway condition characterized by reversible airway obstruction, irreversible airway remodeling, and airway inflammation [1,2]. It is a heterogeneous disorder involving different endotypes and phenotypes [3]. Airway remodeling induced by chronic inflammation is an important characteristic in the pathogenesis of asthma, characterized by subepithelial collagen, goblet cell hyperplasia, and smooth muscle hyperplasia, which is considered to be responsible for persistent airflow obstruction and irreversible airway hyperresponsiveness [4,5]. Preventing accelerated airway remodeling is a critical treatment target of asthma due to the minimal effects of current treatments on airway remodeling [6]. Persistent chronic airway inflammation in asthma induces the conversion of epithelial cells to active mesothelial cells, which is the pathological manifestation of epithelial–mesenchymal transition (EMT). This suggests that EMT is implicated in the progression of subepithelial fibrosis and airway remodeling in asthma [7]. During the process of EMT, epithelial marker E-cadherin is downregulated and mesenchymal marker such as α-SMA and vimentin is upregulated, ultimately resulting in impaired airway barrier function and aggravated airway stenosis [8,9]. Therefore, the possible mechanisms related to the link between airway remodeling and EMT need to be fully clarified.

As a member of the S100 calcium-binding family [10], S100A4 is widely expressed in various types of cells, including macrophages, endothelial cells, lymphocytes, neutrophils, fibroblasts, and smooth muscle cells [11,12]. S100A4 interacts with multiple intracellular targets that regulate cell survival, differentiation, growth, and cytoskeletal dynamics [11]. The S100 proteins including S100A4 can be secreted extracellularly by a range of cell types to affect cellular activities in an autocrine or paracrine manner [11,13]. Current studies on S100A4 have mainly focused on its role in regulating tumorigenesis and cancer metastasis [10]. However, emerging evidence has shown that S100A4, as a candidate gene in allergic condition, is involved in pathological inflammatory conditions, such as experimental autoimmune encephalomyelitis, fibrotic disease, cardiovascular disease, and rheumatoid arthritis [14–17]. S100A4 was found to be elevated in the nasal mucus of allergic individuals [18] and in the sputum of asthmatic patients [19]. High level of S100A4 was found in the lung of mouse models with allergic asthma; additionally, the administration of S100A4-neutralizing antibodies in mice decreased inflammatory cell infiltration and accumulation in the lung and bronchoalveolar lavage fluid (BALF) [19]. Recent reports have indicated that S100A4 activates proinflammatory pathways in airway smooth muscle tissues [20], and it is critical for mast cell activation in mouse models of allergic asthma [21]. Moreover, evidence shows that the S100A4-mediated EMT displays a key role in tumor development and non-tumor pathophysiology [16]. Downregulation of S100A4 attenuates cardiac fibrosis by inhibiting extracellular matrix deposition and α-SMA expression [22,23]. However, whether S100A4 mediates the EMT in asthma remains unclear.

Here, we hypothesized that S100A4 affects airway remodeling by regulating EMT process. To determine this, we used a mouse model of asthma and 16HBE cell line to analyze the effect of S100A4 in asthma. We applied anti‐S100A4 antibody administration *in vivo* or transfection technology to silence S100A4 *in vitro* to observe changes in EMT markers.

## 2 Materials and methods

### 2.1 Animals

Forty male BALB/c mice between 6 and 8 weeks of age (25 ± 2 g) were used in this research. The animals were supplied by Vital River Co. Ltd. (Beijing, China). All mice were healthy, and they were maintained under standard laboratory conditions in a 12 h light/dark cycle at 20 ± 3°C. They were fed with commercial pellets and had free access to water. Prior to the experiment, they were quarantined for 7 days to be acclimatized. Efforts were made to alleviate any suffering of the animals. All experimental protocols were conducted following the institution’s ethical guidelines and were approved by the Ethics Commission of Affiliated Hospital of Jianghan University (Hubei, China).

### 2.2 Asthma sensitization and challenge with ovalbumin (OVA)

**Figure 1:**
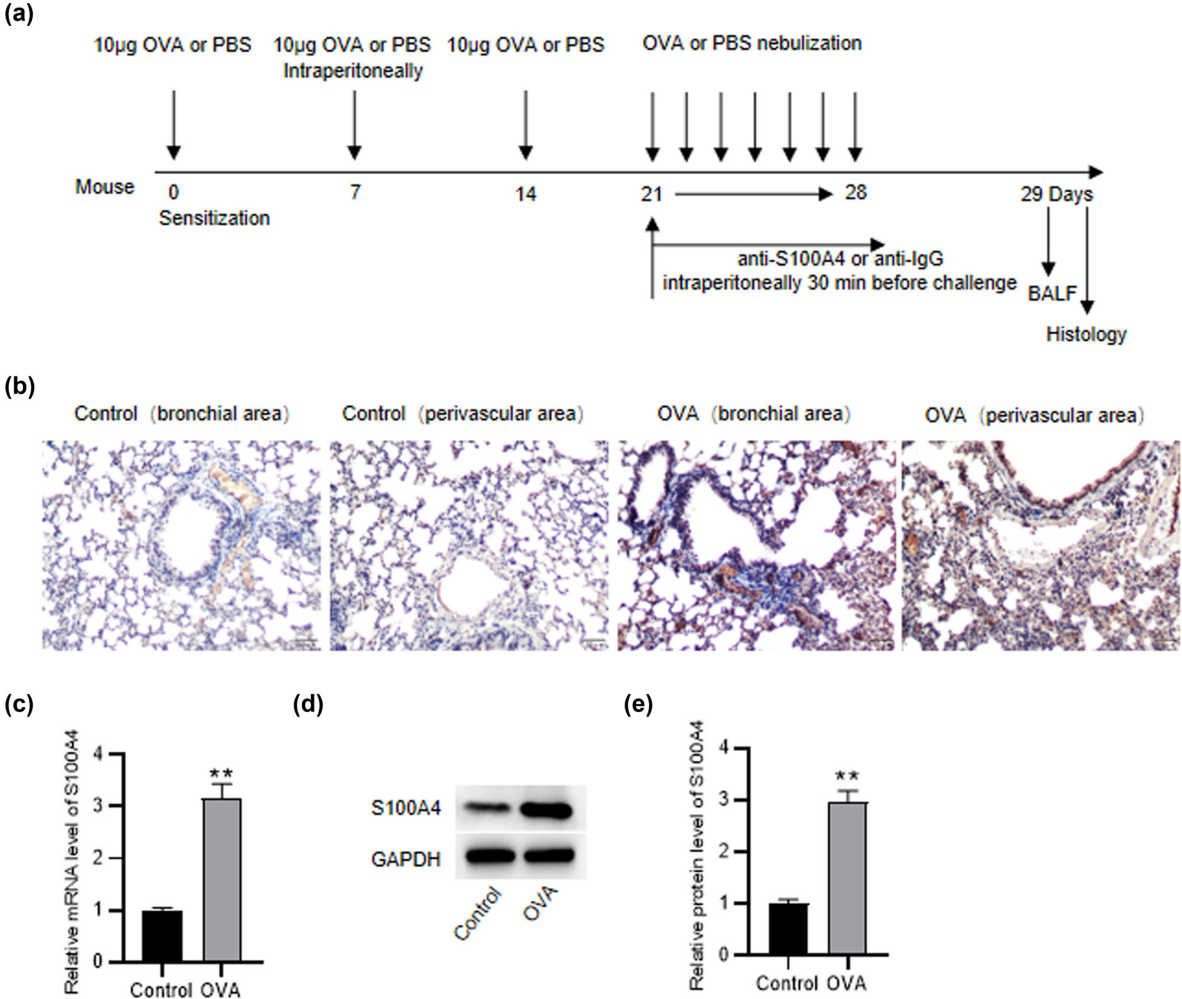
High expression levels of S100A4 in OVA‐induced asthmatic mice. (a) The schematic experimental protocol for drug administration and timeline. (b) Immunohistochemistry analysis of S100A4 in lung tissue sections from OVA‐induced murine asthmatic models. (c) RT-qPCR and (d and e) western blotting analyses of S100A4 expression in lung tissues from OVA mice and PBS control mice. *N* = 10 for each group. ^**^ *p* < 0.1.

Forty male BALB/c mice were assigned into four groups: control, OVA, OVA + isotype antibody, and OVA + anti-S100A4, with ten animals for each group. The asthma model was established as described previously, with minor modifications [24]. Briefly, on days 0, 7, and 14, animals were intraperitoneally sensitized with 200 µl of solution (10 µg OVA emulsified in 1 mg aluminum hydroxide) (Sigma, MO, USA). Between days 21 and 28, animals were challenged with 5% OVA for 30 min daily. Mice in the control received phosphate-buffered saline (PBS) for sensitization and stimulation. Mice were injected intraperitoneally with 100 µg/body of a neutralizing mouse monoclonal IgG1 antibody specific for S100A4 (clone 6B12) or with the corresponding mouse monoclonal IgG1 isotype control (clone MOPC-21, BP0083, BioXcell, NH, USA) at the same concentration for 30 min before the OVA challenge. Isolation and characterization of 6B12 anti-S100A4 mouse monoclonal antibody were described as reported [25]. The schematic experimental protocol for drug administration and timeline is shown in Figure 1a.

### 2.3 Hyperresponsiveness measurement

Twenty‐four hours following the final OVA challenge, airway hyperresponsiveness in response to acetylcholine chloride (3.125, 6.25, 12.5, and 25 mg/mL; Sigma) was detected using a whole-body plethysmography (Buxco Electronics, NY, USA). The detailed procedure was described previously [26].

### 2.4 BALF collection and cell count

Twenty‐four hours following the final OVA challenge, the mice were sacrificed. PBS (100 mL) was used to wash the pulmonary circulation. To collect the BALF, right bronchus was ligated, and left bronchus was flushed three times with 3 mL of PBS via a catheter; the fluid recovery rate was 80%. Cell suspension was centrifuged at 500 g for 10 min at 4°C. Cell pellet was resuspended in 1 mL of normal saline. A total number of cells in 0.05 mL BALF were counted with a hemocytometer (Baxter Diagnostics, USA). The inflammatory cells were dried and stained with Wright-Giemsa (Solarbio, Beijing, China) following the manufacturer’s instructions and were counted based on conventional morphological criteria. Differential cell counts were obtained by counting at least 400 cells per slide. All counts were conducted with blind methods. The remaining BALF supernatants were maintained at −80°C for next use.

### 2.5 ELISA

The concentrations of IL-4, IL-13, TNF-α, and IL-1β in BALF were measured with mouse ELISA kits (R&D Systems, MN, USA) following the manufacturer’s instructions. The optical density for the ELISA was detected using a microplate reader.

### 2.6 Histological staining

Lung tissues were fixed and embedded in paraffin. Then, the blocks were cut into 5 μm-thick sections and subjected to hematoxylin–eosin (H&E) staining according to routine procedures. The severity degree of inflammation was graded referring to previously described scoring criteria [27]. Staining analyses were performed from five randomly selected fields (200× magnification).

### 2.7 RT-qPCR

Total RNA was extracted from lung sections using TRIzol (Invitrogen, USA). The optical density was measured by a spectrophotometer (Bio-Rad, USA) at 260 and 280 nm. RNA was reverse transcribed using commercially reverse transcription kits (Qiagen, Hilden, Germany). Real‐time PCR was performed using ABI PRISM 7500 Real-time PCR System (ABI, CA, USA) and SYBR Premix EX Taq (Takara, Japan). S100A4 expression was determined using the threshold cycle value normalized against GAPDH expression. The used primer sequences are as follows: S100A4: 5′‐ATTTCTGCCAGAGCCGCTTCTACT‐3′ (forward) and 5′‐CAGTTTGTATCCGGCAAACTAGTA‐3′ (reverse), and GAPDH: 5′‐AGGTCGGTGTGAACGGATTTG‐3′ (forward) and 5′‐TGTAGACCATGTAGTTGAGGTCA‐3′ (reverse).

### 2.8 Cell treatment and transfection

Human bronchial epithelial cell line 16HBE (ATCC, USA) was cultured in RPMI 1640 medium (Invitrogen) containing 8% FBS (Gibco, USA). Cells were maintained at 37°C in a humid atmosphere containing 5% CO_2_, and cell growth was observed under a microscope. When cells reached 90% confluence, they were subjected to trypsin digestion. Cells were treated with 100 mg/mL house dust mites (HDMs; STALLERGENES GREER, Lenoir, NC, USA) for 24 h. 16HBE cells were transfected with shRNAs to generate the negative control shRNA (sh-NC) group and S100A4-knockdown (sh-S100A4; GeneChem, Shanghai, China) group. Transfection was performed using Lipofectamine 3000 (Invitrogen) following the manufacturer’s instructions.

### 2.9 Western blotting

Total proteins from cells or tissues were extracted as previously described [28]. Bradford methods (Bio-Rad) were applied to determine protein concentrations. The samples were separated by 8, 10, or 12% SDS-PAGE and electro-transferred to PVDF membranes. After blocking with 5% skim milk for 1.5 h, the membranes were probed with S100A4 (1:1,000; 13018), E-cadherin (1:1,000; 3195), α-SMA (1:1,000; 19245), and vimentin (1:1,000; 5741) overnight at 4°C with GAPDH (1:1,000; 5174) as a loading control. All antibodies are supplied by Cell Signaling Technology. After incubation with corresponding secondary antibodies for 1.5 h at room temperature, an enhanced chemiluminescence reagent (Santa Cruz, CA, USA) was used for color development.

### 2.10 Statistical analysis

All data were processed using SPSS 18.0 (SPSS Inc., Chicago, USA) and are expressed as mean ± standard deviation. Pairwise comparisons were performed using Student’s independent *t*-test and comparisons among multiple groups using one-way ANOVA. The value of *p* < 0.05 indicated statistical significance.

## 3 Results

### 3.1 Increased expression levels of S100A4 in OVA‐induced asthmatic mice

Immunohistochemistry analysis showed weak S100A4 expression in a number of inflammatory cells including macrophages and granulocytes in mice from the control group. However, mice in the asthma mouse model group had moderate expression levels of S100A4 in inflammatory and vascular smooth muscle cells, with prominent S100A4 expression in granulocytes. In addition, S100A4 was weakly stained in a number of epithelial cells in asthmatic mice. Compared to control mice, the number of positive stained S100A4 cells was significantly increased in asthmatic mice (Figure 1b). Additionally, both mRNA and protein levels of S100A4 were notably higher in the lung of asthmatic mice than those of control mice (Figure 1c–e). Forty male BALB/c mice were assigned into four groups: control, OVA, OVA + isotype antibody, and OVA + anti-S100A4, with ten animals for each group.

### 3.2 Treatment with anti‐S100A4 inhibits airway hyperresponsiveness and inflammation in OVA-challenged mouse models

**Figure 2:**
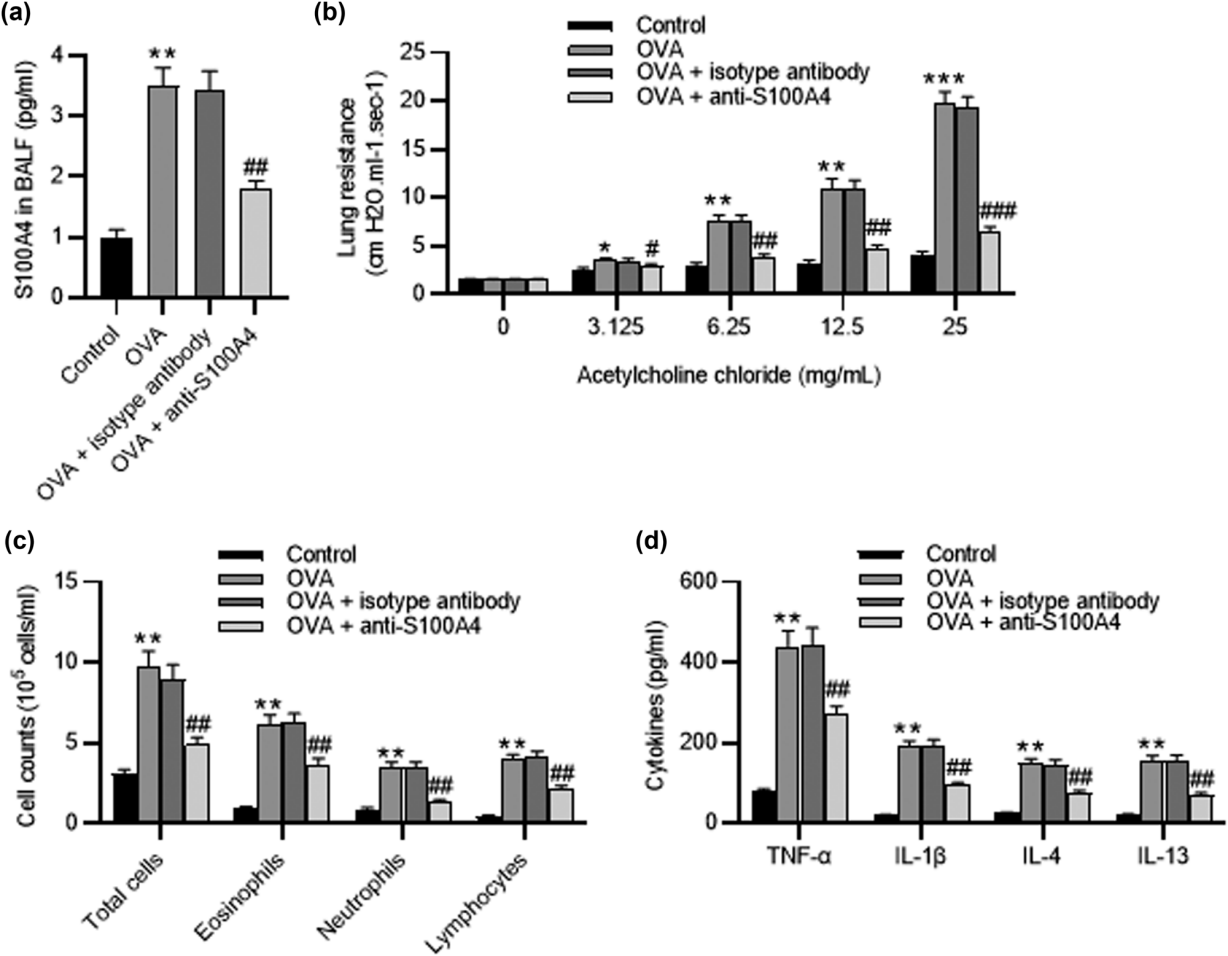
Treatment with anti‐S100A4 inhibits airway hyperresponsiveness and inflammation in BALF from OVA-challenged mouse models. Forty male BALB/c mice were assigned into four groups: control, OVA, OVA + isotype antibody, and OVA + anti-S100A4. (a) ELISA of S100A4 levels in BALF. (b) Airway hyperresponsiveness in each group. (c) The number of total inflammatory cells and cellular components in BALF. (d) ELISA of TNF-α, IL-1β, IL-4, and IL-13 levels in BALF. *N* = 10 for each group. ^**^ *p* < 0.1 vs the control group; ^##^ *p* < 0.1 vs the OVA group.

ELISA showed that OVA-challenged mice had a higher level of S100A4 in BALF than control mice. Anti‐S100A4 administration markedly reduced S100A4 expression level in BALF (Figure 2a). We evaluated the effect of anti-S100A4 on airway hyperresponsiveness in response to acetylcholine chloride. Baseline airway resistance had no significant differences among all groups. Acetylcholine chloride at doses increasing progressively significantly increased lung resistance in OVA mice compared to control mice. However, anti‐S100A4 administration resulted in a marked reduction of lung resistance in OVA mice (Figure 2b), suggesting that anti‐S100A4 inhibited airway hyperresponsiveness. Following the OVA challenge, the increased number of total cells, as well as neutrophils, eosinophils, and lymphocytes in BALF, was found (Figure 2c). ELISA showed upregulated levels of TNF-α, IL-1β, IL-4, and IL-13 in BALF induced by OVA (Figure 2d). However, the number of inflammatory cells and inflammatory cytokines was significantly reduced after anti‐S100A4 administration (Figure 2c and d). This suggested that anti‐S100A4 suppressed airway inflammation in OVA mice.

### 3.3 Treatment with anti‐S100A4 alleviates airway inflammation in OVA-challenged mouse models

**Figure 3:**
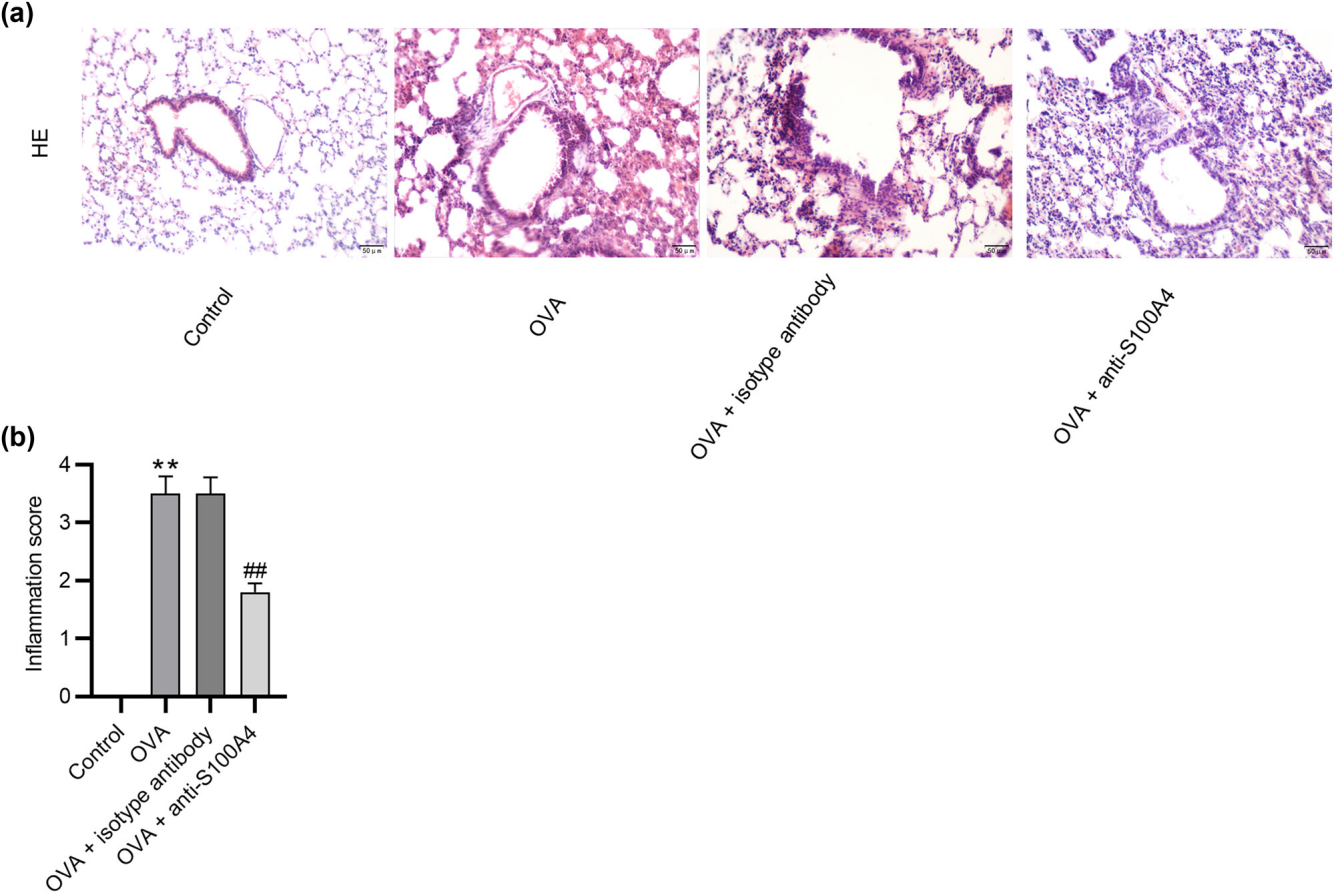
Treatment with anti‐S100A4 alleviates airway inflammation in OVA-challenged mouse models. (a) Infiltration of inflammatory cells in lung tissues was evaluated using H&E staining. Magnification: 200 ×. Scale bar: 50 µm. (b) Inflammation score. *N* = 10 for each group. ^**^ *p* < 0.1 vs the control group; ^##^ *p* < 0.1 vs the OVA group.

Airway inflammation was further assessed using histopathological analyses of lung tissues. H&E staining showed that OVA-challenged mice presented massive peribronchial and perivascular infiltration of inflammatory cells, accompanied by increased inflammation score compared with control mice. However, anti‐S100A4 administration decreased infiltration of inflammatory cells and inflammation score caused by OVA (Figure 3a and b).

### 3.4 Treatment with anti‐S100A4 inhibits EMT in asthmatic mice

**Figure 4:**
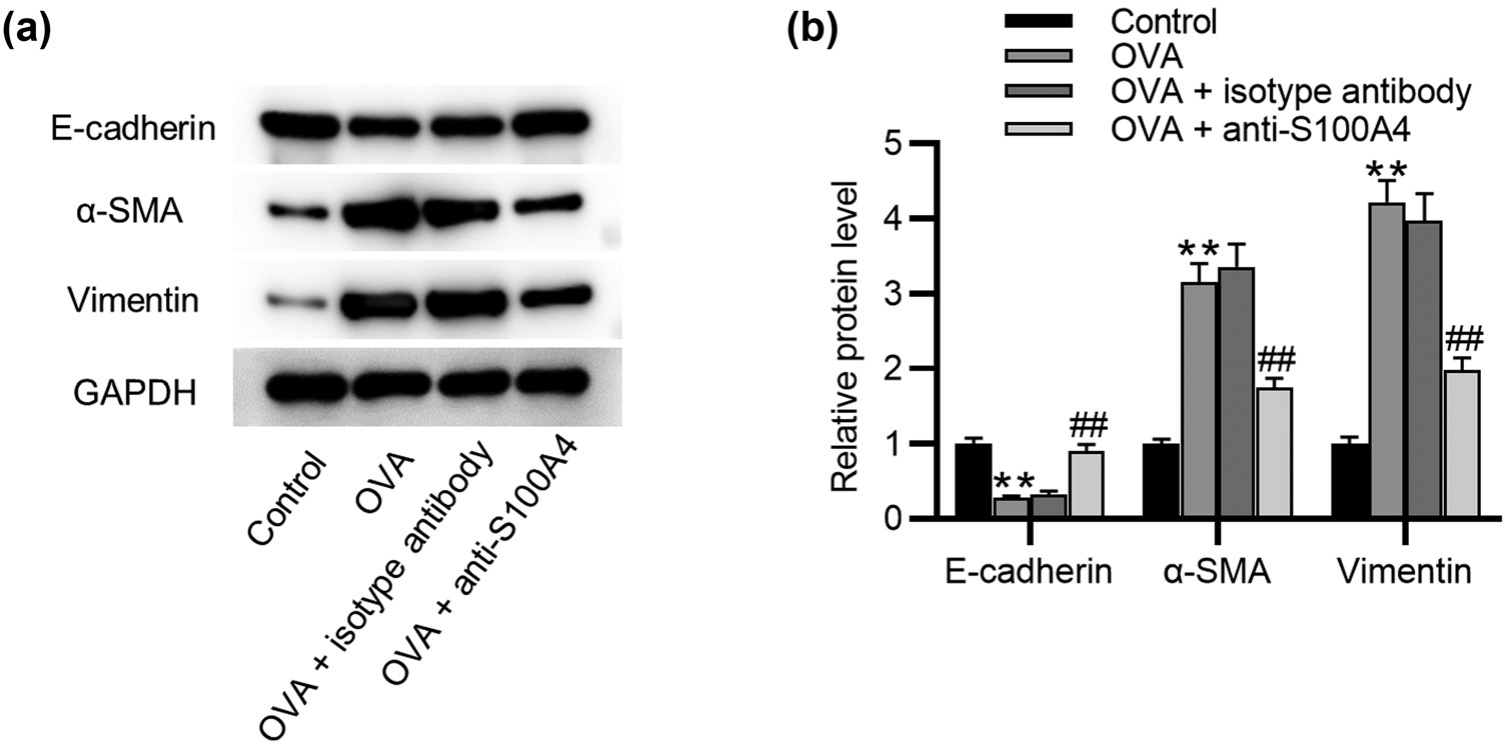
Treatment with anti‐S100A4 inhibits EMT in asthmatic mice. (a and b) Western blotting analysis of E‑cadherin, α-SMA, and vimentin in lung tissues. *N* = 10 for each group. ^**^ *p* < 0.1 vs the control group; ^##^ *p* < 0.1 vs the OVA group.

EMT plays a key role in airway narrowing and obstruction caused by airway remodeling. As shown by western blotting in Figure 4a and b, a significant reduction of the E-cadherin level and an elevation of the α-SMA and vimentin levels were detected in the lung of OVA mice. Following administration of anti-S100A4, the E-cadherin level was upregulated, while the α-SMA and vimentin levels were downregulated, suggesting that the OVA-induced EMT process was inhibited by anti-S100A4.

### 3.5 S100A4 downregulation inhibits EMT in HDM-stimulated 16HBE cells

**Figure 5:**
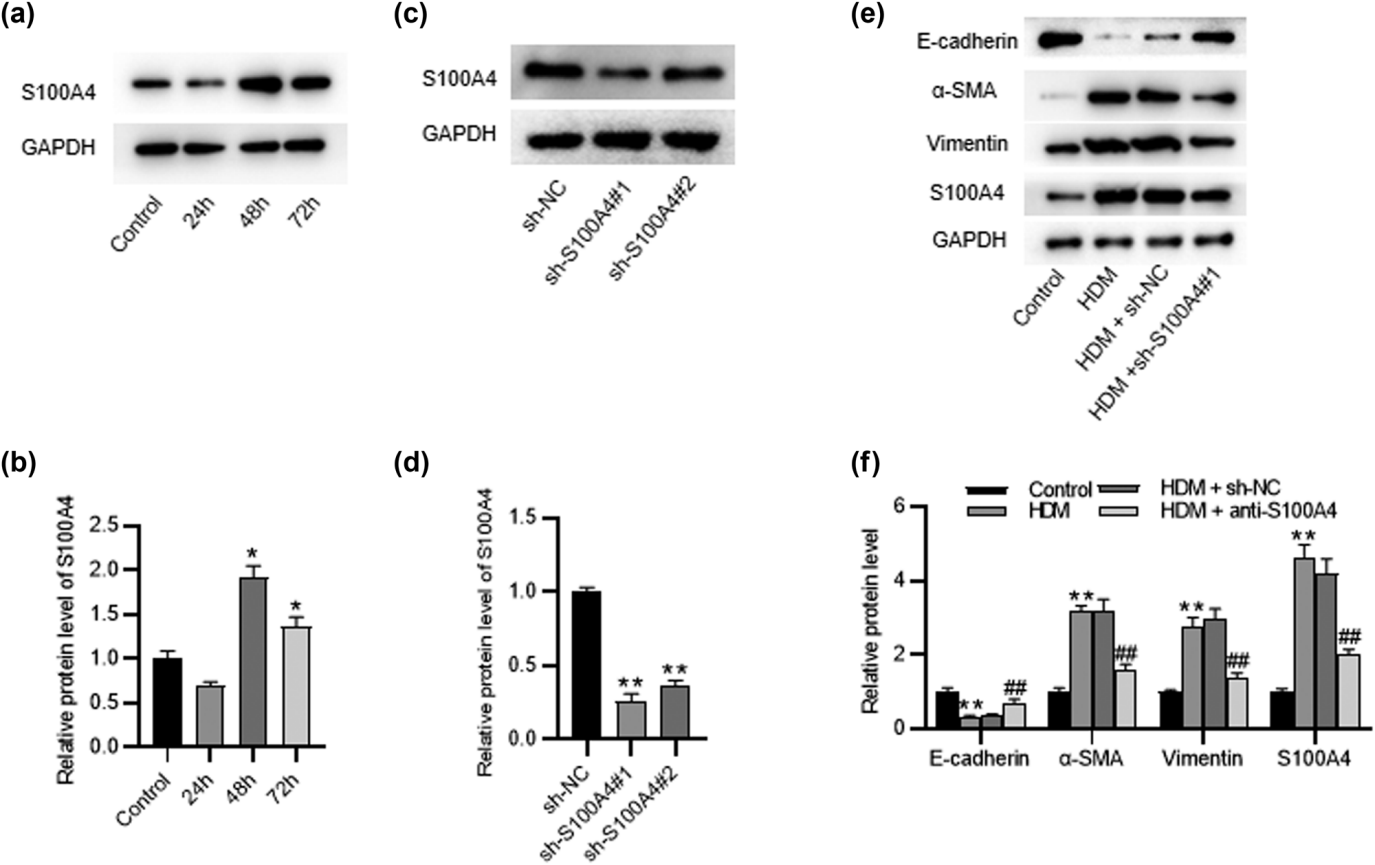
S100A4 downregulation inhibits EMT in HDM-stimulated 16HBE cells. (a and b) Western blotting analysis of S100A4 level in 16HBE cells after treatment with 100 ng/mL HDM for 24, 48, and 72 h. (c and d) Western blotting analysis of S100A4 level in 16HBE cells transfected with sh-S100A4 or control. (e and f) Western blotting of E‑cadherin, α-SMA, vimentin, and S100A4 levels in HDM-stimulated 16HBE cells transfected with sh-S100A4 or control. ^*^ *p* < 0.5, ^**^ *p* < 0.1 vs the control or sh-NC group; ^##^ *p* < 0.1 vs the HDM group.

The *in vitro* effect of S100A4 on EMT was evaluated in HDM-stimulated 16HBE cells. After 16HBE cells were stimulated with 100 ng/mL HDM for 24, 48, and 72 h, western blotting examined the S100A4 level, showing that the S100A4 level had the most significant upregulation at 48 h (Figure 5a and b). 16HBE cells were transfected with sh-NC or sh-S100A4. The protein expression level of S100A4 was lower in cells transfected with sh-S100A4#1 than those with sh-S100A4#2 (Figure 5c and d); therefore, sh-S100A4#1 was applied in subsequent experiments. Western blotting showed that the E-cadherin level was downregulated, while the α-SMA and vimentin levels were upregulated in HDM-stimulated 16HBE cells. As expected, transfection of sh-S100A4#1 markedly upregulated E-cadherin level and downregulated α-SMA and vimentin levels in HDM-stimulated 16HBE cells (Figure 5e and F), suggesting that S100A4 downregulation inhibited EMT in HDM-stimulated 16HBE cells.

## 4 Discussion

Asthma is a chronic airway condition with complex predisposing factors and pathogenesis. Airway remodeling in asthmatic patients is intractable under the current treatment. Reduction of epithelial proteins and elevation of mesenchymal proteins are pathological manifestations of EMT, which occur during the process of airway remodeling. In this study, BALB/c mice were subjected to OVA sanitization to establish animal models of asthma. Based on this model, airway hyperreactivity, airway inflammation, and EMT characteristics were examined in asthmatic mice. We detected elevated levels of S100A4 in the lung and BALF of OVA-challenged mice, suggesting that S100A4 may be associated with the pathogenesis of asthma. The treatment effect of S100A4 on OVA-induced asthma was investigated.

S100A4 has been implicated in the development of several lung diseases. Expression of S100A4 was significantly increased in a dose‐ and time‐dependent manner in lung tissues of bleomycin‐induced murine models for pulmonary fibrosis and localized mainly in lung fibroblasts [29]. S100A4, used as a marker for EMT, stained weakly in a number of biopsy epithelial cells from patients with stable lung allografts [15]. S100A4 mRNA and protein expression were elevated in pulmonary arteries of mice treated with hypoxia. S100A4 protein was specifically localized in vascular smooth muscle cells and fibroblasts as demonstrated by immunohistochemistry analysis [30]. It is unclear what types of cells in the lungs are responsible for S100A4 secretion; however, cell types like pulmonary artery smooth muscle cells [31], fibroblasts [30], and several cancer cells [32] have been identified to secrete S100A4. S100A4 immuno‐staining in lung tissues in our study showed that S100A4‐stained cells were mainly infiltrating inflammatory cells, especially granulocyte and vascular smooth muscle cells. Furthermore, the number of S100A4‐positive cells in the interstitial and peri‐vascular regions was significantly increased in OVA‐induced asthmatic mice compared to control mice. This suggested that inflammatory cells, especially granulocytes and vascular smooth muscle cells, are potential sources of S100A4 in the airway and lungs. However, further studies need to be performed to confirm the cell types responsible for S100A4 secretion and determine the mechanism underlying the secretion process.

In this study, OVA-challenged mice exhibited asthma-like symptoms. Airway hyperreactivity is a main feature of asthma. Acetylcholine chloride at doses increasing progressively significantly increased lung resistance in OVA mice compared to control mice, suggesting that airway hyperreactivity occurred in OVA-challenged mice. Airway inflammation is considered a key trigger for airway hyperreactivity. Proinflammatory cytokines IL-4 and IL-13 secreted by Th2 are closely associated with mucus secretion, eosinophil activation, and airway remodeling in asthma [33,34]. Studies demonstrate that controlling Th2-type asthma is effective via the preparation of anti-IL-4/IL-13 antibodies [35]. S100A4 has been shown to suppress airway inflammation in chronic asthmatic mouse models [19]. In this study, treatment with anti‐S100A4 antibody significantly inhibited Th2-mediated airway hyperreactivity and reduced the production of IL-4 and IL-13. Inflammatory cell infiltration around the airway also aggravates the progression of asthma [36]. Eosinophil gathering around the bronchus and release of cytotoxic granule protein contribute to collagen deposition and epithelial cell injury [33], which is required for airway remodeling. The cytokines (such as IL-1β and TNF-α) are also involved in promoting the allergic response [37]. Therefore, there is ongoing research on how to prevent the release of inflammatory factors [38]. Here, the administration of anti‐S100A4 antibody *in vivo* alleviated airway remodeling by reducing airway hyperreactivity and inflammation. Extracellular S100A4 can affect numerous cell types by binding to their receptors. Epidermal growth factor receptor (EGFR) [39] and the receptor for advanced glycation end products (RAGE) [40] have been shown to be the extracellular receptors for S100A4. S100A4 can enhance EGFR/ErbB2 receptor signaling pathway to induce cell proliferation and promote tumor progression [39]. In addition, extracellular S100A4 can also interact with RAGE to increase plasmin levels and thus induce tube formation in endothelial cells [40]. More importantly, S100A4 plays a crucial role in leukocyte migration, which has been associated with the pathogenesis of inflammatory diseases. S100A4 can induce the secretion of cytokines, particularly eotaxin‐2 and GM‐CSF from T lymphocytes [41]. We hypothesized that the potential role of S100A4 in asthmatic airway inflammation may be partially attributed to its ability to promote the infiltration of inflammatory cells.

E-cadherin, vimentin, and α-SMA proteins are key markers in the process of EMT [42]. Loss of epithelial cell adhesion protein E-cadherin causes the dysfunction of epithelial barrier, resulting in the loss of structural stability and polarity of the bronchial epithelium, which is required for the migration of bronchial epithelial cells. Increased vimentin and α-SMA expression alters the composition of cytoskeletal proteins and thereby contributes to the epithelial cubic‑shaped cells into fiber‑like cells. These cells thus obtain the potential of migration [43]. It has been suggested that low E-cadherin expression in patients with asthma is related to the dysfunction of airway barrier and the development of airway remodeling [44]. A study reported decreased expression of E-cadherin and elevated expression of vimentin in the airway epithelium from dust mite-induced asthmatic mice [45]. We also found that E-cadherin was downregulated and vimentin and α-SMA were upregulated in OVA-challenged mice and in HDM-stimulated epithelial cells. Although the relationship between S100A4 and airway inflammation was elucidated, whether it affects EMT in asthma remains unknown. S100A4 drives EMT in chronic sinusitis mucosal epithelial cells and accelerates nasal mucosa tissue remodeling [46]. S100A4 silencing inhibits TGF-β-induced EMT in pleural mesothelial cells [47]. In the current study, anti-S100A4 administration downregulated vimentin and α-SMA levels and upregulated E-cadherin level in OVA-challenged mice. S100A4 downregulation also inhibited EMT process in HDM-stimulated 16HBE cells. These findings suggested that silencing S100A4 may be a possible mechanism to prevent the bronchial epithelium from fibrosis in asthma. However, we only investigated the effects of intracellular S100A4 on EMT process in human bronchial epithelial cells. We plan to investigate whether extracellular S100A4 exerts the effects on EMT *in vitro* in future studies. This will support our data in mouse models.

## 5 Conclusion

In summary, we verified that S100A4 was elevated in the bronchial epithelium from mouse models of airway remodeling and in human bronchial epithelial cell line stimulated by HDM. Additionally, intracellular S100A4 blockage attenuated airway remodeling by the inhibition of airway inflammation and EMT process, suggesting that S100A4-antibody therapy may have clinical applicability in controlling airway remodeling in patients with asthma. However, further investigations should be conducted to elucidate the mechanisms of S100A4 in asthma.
